# The release of petroleum hydrocarbons from a saline-sodic soil by the new biosurfactant-producing strain of *Bacillus* sp.

**DOI:** 10.1038/s41598-022-24321-3

**Published:** 2022-11-17

**Authors:** Sahar Kalvandi, Hamidreza Garousin, Ahmad Ail Pourbabaee, Mohsen Farahbakhsh

**Affiliations:** Biology and Biotechnology Lab, Department of Soil Science, University College of Agriculture and Natural Resources, University of Tehran, Karaj, Iran

**Keywords:** Biotechnology, Microbiology, Environmental sciences

## Abstract

Adsorption of old-aged petroleum hydrocarbons to the soil solid phase, which causes biosurfactant loss of performance, is among the limiting factors for the remediation of the saline-sodic soils contaminated with petroleum. Therefore, to find a functional biosurfactant in oil-contaminated saline-sodic soils, the efficiency of 39 bacteria isolated from petroleum-contaminated soils was evaluated. The strains were cultured in the Bushnell–Haas medium, and the produced biosurfactants and bioemulsifiers in this medium were extracted using chloroform/methanol and ethyl acetate extraction methods, respectively. Their partial purification was performed by column chromatography, and eventually, their performance in releasing TPH from the contaminated soil was evaluated. The soil test results revealed that the highest TPH releases due to the effects of the biosurfactants and bioemulsifier produced from SHA302, SH21, and SH72 isolates were 42.4% ± 0.2, 21.6% ± 0.15 and 24.3% ± 0.91, respectively. Based on the 16S rRNA gene sequence, the SHA302 strain showed 93.98% phylogenetic similarity with *Bacillus pumilus* strain ATCC 7061. The Fourier transform infrared spectroscopy and thin-layer chromatography results proved that the biosurfactants produced by isolates SHA302, SH21 and SH72 showed lipopeptide, glycolipoprotein and glycoprotein natures, respectively. The performance of the biosurfactant produced by SHA302 isolate indicated that it could be used as a good candidate for releasing TPH from saline-sodic soils with old contamination and facilitating the degradation of hydrocarbons.

## Introduction

Soil contamination with petroleum hydrocarbons is one of the significant environmental concerns in the world^1^. The low solubility of some petroleum hydrocarbons makes them resistant to the bioremediation process and, thereby, reduces their solubility for microorganisms^2^. Petroleum hydrocarbons can be removed from soil using different mechanisms such as leaching, evaporation and degradation by native soil microorganisms. However, these mechanisms fail to remove some petroleum hydrocarbons due to their precipitation in the soil solid phase^3^. An increase in contact time between petroleum hydrocarbons and soil particles leads to a reduction in the availability of these contaminants, which is called “ageing”^4^. The main mechanisms involved in the ageing process are adsorption and diffusion between petroleum hydrocarbons and the soil solid phase^5^.

One of the most promising solutions to increase the limited bioavailability of contaminants is the use of those biosurfactants which play a vital role in the desorption of organic contaminants from the surface of soil particles^6^. Chemical surfactants are toxic, difficult to degrade, and harmful to the environment. Compared to chemical surfactants, biosurfactants have more comparative advantages, such as low toxicity, biodegradability, and resistance to extreme acidity and temperature changes^7^. On the other hand, biosurfactant production capacity is higher due to the availability of many cheap substrates^7,8^. Biosurfactants surge the contaminants present in the soil soluble phase and, thus, increase their availability to the microorganisms which can degrade them^6^. Biosurfactants enhance the bioremediation of hydrocarbons using two mechanisms: (1) increasing the bioavailability of pollutants and (2) increasing the cell surface hydrophilicity^9^. Biosurfactants increase the surface of insoluble compounds by reducing both surface tension (ST) and interfacial tension, leading to increased mobility and hydrocarbons bioavailability. The addition of biosurfactants increases petroleum hydrocarbons biodegradation by increasing mobilization, solubilization and/or emulsification^10^. The mobilization process occurs at concentrations below the surfactant’s critical micelles concentration (CMC), and the solubilization process takes place at concentrations higher than the surfactant’s CMC^11^. In the adsorption of biosurfactants to the solid soil particles, the mobilization process depends on the biosurfactants charge^12^. However, adsorption to the soil may reduce their efficiency^12^. The contaminant solubilization process is associated with the formation of surfactant micelles.

The hydrophobic tails of surfactant molecules link to the inner part of the micelle, while their hydrophilic heads are located in the outer part of the micelle and are in contact with the solution phase. The solubilization process begins with the penetration of petroleum hydrocarbons into the micelles. This is an effective tactic by which biosurfactants increase the solubility of hydrophobic compounds in the soil^13,14^. Sodic and saline-sodic soils cover more than 50% of salt-affected lands, and most oil-related industries are located in the areas with saline soils; however, the study of the release of petroleum contaminants from saline-sodic soils seems to be essential^15^. The present study aimed to screen the bacterial isolates capable of producing biosurfactants. The identified biosurfactants were subjected to various tests, such as salinity stability, emulsion structure, antibacterial properties and adsorption to the soil, to find the efficient biosurfactant in releasing TPH from the saline-sodic contaminated soil.

To the best of the author’s knowledge, no study has been conducted on the petroleum hydrocarbons released from a highly old-aged petroleum-contaminated saline-sodic soil using biosurfactants.

## Results and discussion

### Screening of surfactant-producing isolates

The initial screening test of the isolates showed that among the 39 isolates from petroleum-contaminated soils, only seven isolates (SH21, SH41, SH42, SHA302, SH83, SH103 and SH72) had an ability to produce surfactant. All the seven isolates, except for SH72, could reduce ST to less than 40 mN/m (Table 1). Reference^16^ reported that the strains with an ability to reduce ST to less than 40 mN/m could be considered potentially biosurfactant-producing bacteria. In the screening process of surfactant-producing bacteria, Ref.^17^ selected the strains capable of reducing ST to less than 40 mN/m for further investigation. Although the SH72 isolate did not significantly reduce ST, it showed high emulsifying activity and was selected as a bioemulsifier for further studies. Bioemulsifiers are high molecular weight biopolymers or extracellular polysaccharides that, similar to biosurfactants, can emulsify immiscible compounds, such as hydrocarbons or other hydrophobic compounds, even at low concentrations. However, the effectiveness of bioemulsifiers in reducing ST is not significant^18^. The statistical analyses in this study showed that ST had a significant negative correlation with the oil spreading test (r = − 0.945, P ≤ 0.0001), emulsification (r = − 0.874, P ≤ 0.0001), foaming (r = − 0.766, P ≤ 0.0001), and blood hemolysis (r = − 0.784, P ≤ 0.0001). Due to their higher correlations, oil spreading and ST tests were considered the main tests in selecting isolates. Reference^19^ showed that the increase in halo diameter in the oil spreading test was proportional to the decrease in ST, and there was a negative correlation (r = − 0.959) between these two tests. In an investigation^20^ presented that ST showed a negative correlation with both oil spreading test (r = − 0.971) and emulsification test (r = − 0.987).

**Table 1:** Characteristics of produced surfactants by seven bacterial isolates.

Isolates	Emulsification test (%)	Foaming test (%)	Surface tension (mN/m)	Oil spreading	Blood analysis
SH21	52.7 ± 0.25	0.21 ± 25.2	32.8 ± 0.26	+++	+++
SH41	25.4 ± 0.11	48.35 ± 0.25	36 ± 0.24	++	+++
SH42	18.53 ± 0.35	5.4 ± 0.24	38.4 ± 0.25	++	+
SHA302	40.3 ± 0.12	42.4 ± 0.21	36.5 ± 0.23	+++	+++
SH72	85.5 ± 0.1	–	48.6 ± 0.28	+	+++
SH83	58.8 ± 0.16	9.4 ± 0.12	30.47 ± 0.33	+++	++
SH103	22.5 ± 0.24	15.6 ± 0.21	28.4 ± 0.17	+++	+++

Oil Spreading Test (cm): ( +) mean of the halo diameter between 0.5 and 0.9, (++) between 1 and 1.5, (+++) between 1.5 and 2.1, (++++) between 2.1 and 3. Blood hemolysis test (cm): (–) without hemolysis, (+) incomplete hemolysis, (++) complete hemolysis with diameter < 1, (+++) complete hemolysis with diameter between 1 and 3, (++++) complete hemolysis with diameter > 3. The initial ST of the culture medium was equal to 70.5 mN/m.

### Evaluation of salinity stability

**Figure 1:**
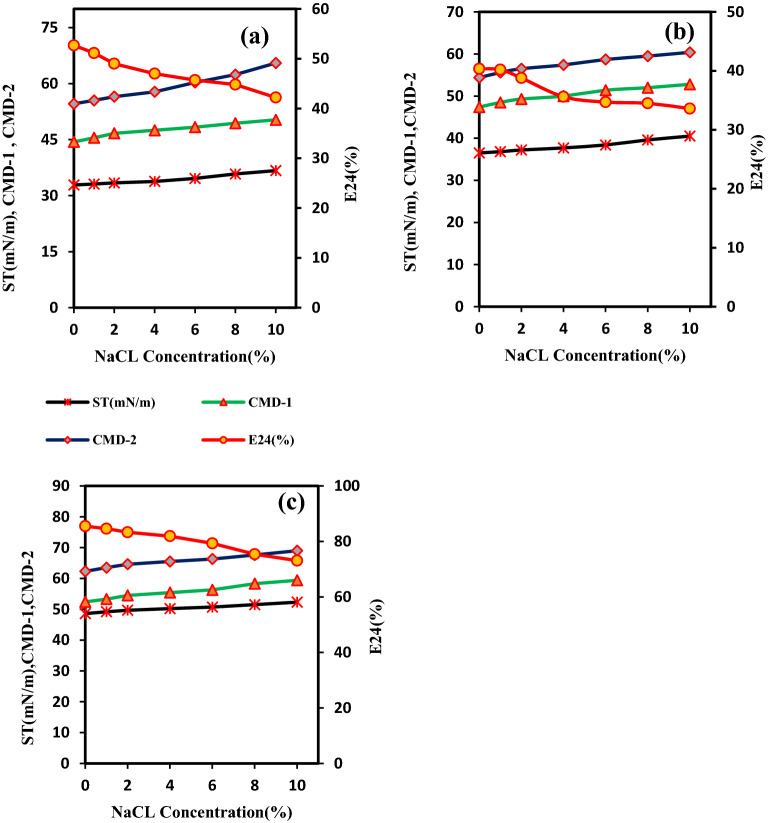
Evaluation of salinity stability (0–10%) in three different surfactants produced by (**a**) SH21, (**b**) SHA302 and, (**c**) SH72. *ST* surface tension, *E24* emulsification test, *CMD*^*−1*^* and CMD*^*−2*^ critical Micelle dilution of broth diluted 10 and 100 times, respectively.

The stability of biosurfactants/bioemulsifiers to salinity is an essential factor. Considering the type of contaminated soils and the presence of salinity, it was not feasible to use any surfactant for remedying the old-contaminated saline-sodic soils. Salinity stability was investigated as a secondary screening for more effective screening of the biosurfactant-producing isolates. The results showed that among the seven isolates selected, the biosurfactants produced by SHA302, SH21, and SH72 isolates (in CFC) showed significant salinity stability changes compared to the other four isolates (SH41, SH42, SH83, and SH103). Therefore, the four isolates were excluded from further investigation. In the supernatant containing the biosurfactant produced by SH21 isolate, the amount of ST and emulsifying activity changed from 32.8 (initial) to 36.7 mN/m (10% salinity) and from 52.7 (initial) to 42.2% (10% salinity), respectively. The changes of CMD^−1^ and CMD^−2^, as a function of ST, varied from 44.4 to 50.3 (mN/m) and from 54.6 to 65.5 (mN/m), respectively (Fig. 1a). In the supernatant containing the biosurfactant produced from SHA302 isolate, ST increased from 36.5 to 40.5 (mN/m), and the emulsifying activity decreased from 40.3 to 33.6%. Moreover, CMD^−1^ and CMD^−2^ increased from 47.3 to 52.7 (mN/m) and from 54.3 to 60.4 (mN/m), respectively, with the increase of salinity (Fig. 1b). In the supernatant containing the biosurfactant produced from SH72 isolate, ST raised from 48.6 to 52.2 (mN/m), and the emulsifying activity decreased from 85.5 to 73.1% (Fig. 1c). The results of this study were in agreement with the results reported by Refs.^21–25^. Reference^26^ showed that, with the increase of salt concentration, the amount of ST increased due to the formation of bipolar interaction with water. They concluded that bipolar interaction was more robust than gas-salt phase interaction, and it could prevent the entry of solute molecules to the surface (liquid–gas). Reference^27^ demonstrated that the decreasing trend in emulsifying activity was due to demulsifying properties of surfactants.

### Extraction of biosurfactants and bioemulsifier

Extraction and purification of biosurfactants and bioemulsifiers account for about 60% of total production costs, preventing commercialization on a big scale. Fortunately, purification of these compounds is not crucial in agriculture as well as bioremediation and leads to reducing many production costs. The biosurfactants extraction methods vary according to their ionic charge, solubility and/or their extracellular or intracellular nature^28–30^. The statistical results showed that in all the three extraction methods (ethyl acetate, methanol and acid precipitation), there was a significant difference in the amount of the biosurfactant produced from SH21 and SHA302 at the probability level of 5% (P ≤ 0.0001). Compared to other extraction methods, the chloroform/methanol method led to the highest amount of biosurfactants produced by SHA302 and SH21 isolates (Fig. S1a,b). This result could be due to the hydrophobic/hydrophilic equilibrium of the chloroform/methanol solvent system^31^. Chloroform is a popular solvent, especially for medium-polar lipids, and when combined with methanol, it becomes a universal extracting solvent^32^.

Moreover, the statistical results of the emulsification of SH72 isolate indicated that the extraction with ethyl acetate led to a significantly different outcome compared to other extraction methods (Table S2, Fig. S1c). Standard techniques for extracting bioemulsifiers include chloroform/methanol, dichloromethane/methanol, ethyl acetate, and tetrahydrofuran^33,34^. However, the solvent type depends on the type of microbial strain and the type of produced compound^35^. The hydrophobicity of proteins is determined based on the hydrophobicity of amino acids, and their degree of hydrophobicity depends on the surface area and the polarity degree of the molecule^36,37^. Due to the glycoprotein structure of this bioemulsifier, it is expected that ethyl acetate, with a lower degree of polarity than ethanol and acetone, is more efficient in extracting proteins (assuming the presence of hydrophobic amino acids in the peptide chain).

Generally, there was a significant difference between extraction with organic solvents using the acid precipitation method and extraction with cheap salts. As low-cost alternative methods for extracting biological compounds, we can use the acid extraction method for biosurfactants and the mineral extraction method (zinc sulfate and ammonium sulfate) for bioemulsifiers on a larger scale to reduce the cost and use of poisonous solvents. Extraction of biosurfactants with organic solvents, acids and salts by dissolving into organic solvents, insolubilization of biosurfactant at low pH, and inducing salting-out phenomenon, respectively, lead to the precipitation of biosurfactants in the solution^38^.

### CMC and observation of emulsion structure by optical microscope

**Figure 2:**
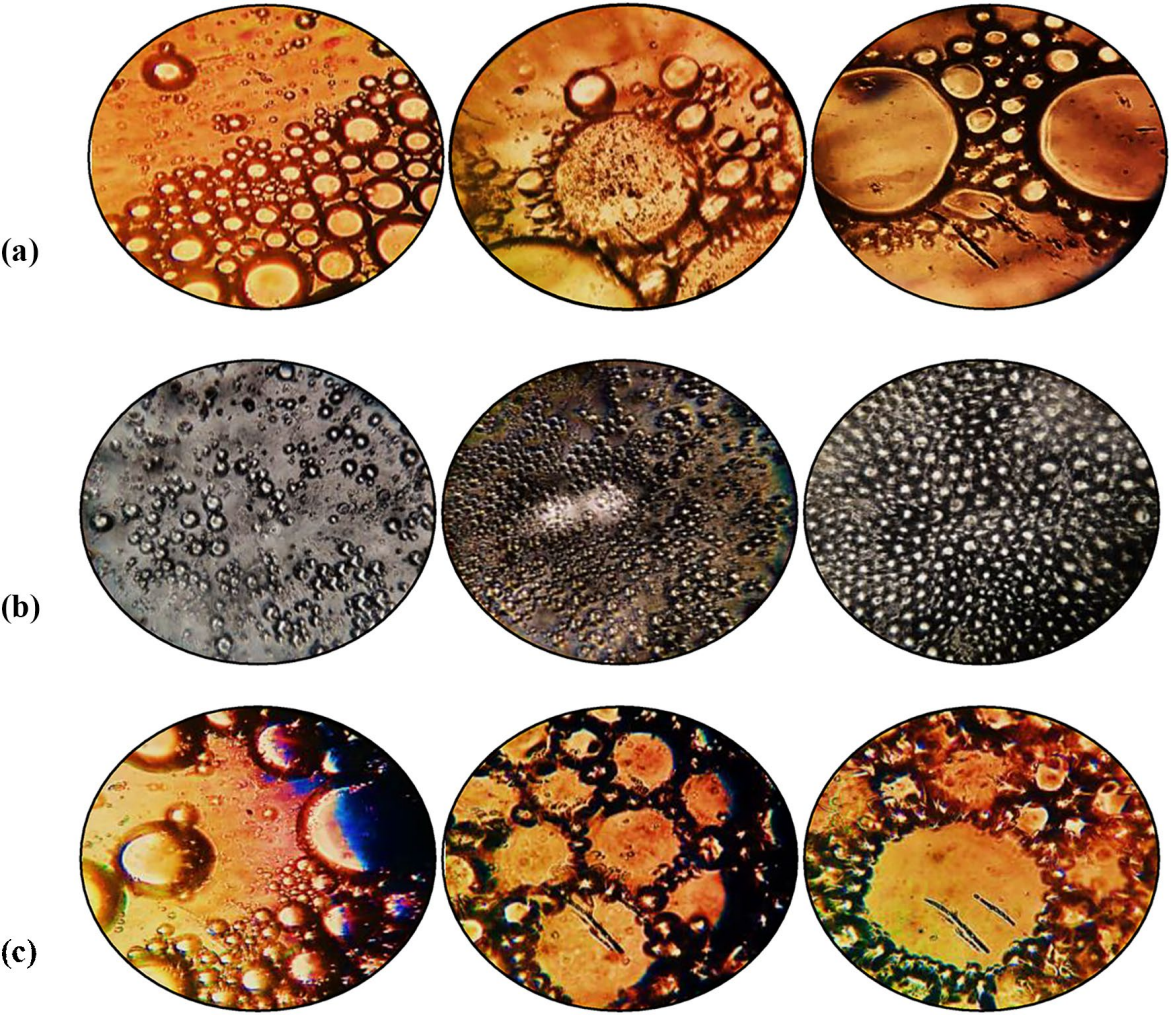
Emulsion structure of produced biosurfactants and bioemulsifier by light optical microscope (× 40) in different concentrations of 1.2 × CMC, CMC, and 2 × CMC (in order from left to right) for isolates (**a**) SH21, (**b**) SHA302, and (**c**) SH72.

CMCs for SH21, SHA302 and SH72 isolates were considered to be 195, 115 and 58 (mg/l), respectively. The stability of emulsion refers to its ability to resist changes in physical and chemical properties over time^39^. Mechanisms that lead to emulsion instability are gravitational separation (cream/sediment), Ostwald ripening, coalescence, flocculation, and phase inversion. The stability of emulsions is affected by the properties of their droplets. Essential characteristics of the droplets include concentration, size, charge, interactions, and geological behaviour^39,40^. In the surfactant produced by SH21 isolate, the particles displayed a spherical structure, and their size approximately increased with the increase in concentration. At concentrations of 1.2 × CMC, 2 × CMC, and CMC, the particle diameters were 1–55, 1–138, and 1–112 µ, respectively (Fig. 2a)^41^. This means that the surfactant concentration and the emulsion stability are directly related to each other.

In the surfactant produced by strain SHA302, the surfactant particles showed a diameter of approximately 1–15 µm at the three concentrations and a constant trend in size and shape (Fig. 2b). In the emulsifier produced by strain SH72, the particle size increased with the increase of concentration. Thus, at different concentrations of 1/2 × CMC, CMC, and 2 × CMC, the particle sizes were in the range of 1–102, 1–142, and 1–182 µm, respectively (Fig. 2c). The droplets distribution in a stable emulsion is homogeneous with a relatively small size. A flocculation emulsion is formed when larger droplets are placed close to each other without merging into other bigger droplets. If small and large droplets exist simultaneously, joining may be induced by Ostwald ripening or coalescence, leading to emulsion instability^42^. The emulsion droplets obtained from the SHA302 isolate had a homogeneous distribution, and their sizes did not significantly change with the increase in the surfactant concentration. Therefore, the emulsion obtained from this surfactant was expected to be more stable than the emulsion derived from the other two surfactants.

### Identification of biosurfactants and bioemulsifier by TLC and FTIR

TLC did identify the three biosurfactants. Staining with ninhydrin, molisch, and iodine reagents revealed the presence of amino acids, sugars, and lipids in the structure of the surfactant produced by SH21 isolate, respectively (Fig. S2c–e). The surfactant produced by the SHA302 isolate did not show any spot during staining with molisch reagent. However, staining with ninhydrin and iodine reagents with different retention factors detected the spots in this surfactant (Fig. S2a,b). For the emulsifier produced by SH72 isolate, the spots appeared during staining with ninhydrin and molisch reagents rather than iodine reagent (Fig. S2f,g). According to the FTIR results for the biosurfactants and the bioemulsifier (Fig. S3), the adsorption band in the range of 3500–3300 cm^−1^ represented N–H bond in a peptide component. The adsorption band in the range of 1000–1200 cm^−1^ represented the C–C and C–O–P functional groups in oligo and polysaccharides (starches)^43^. The adsorption band in the range of 1763–1712 cm^−1^ corresponded to the C=O functional group in cellulose–fatty acids.

stretching of esters, and the adsorption band in the range of 1481–1585 cm^−1^ indicated the protein amide II band mainly N–H bending and C–N stretching in the two biosurfactants produced by SH21, and SHA302 isolates^44^.

In the surfactant produced by SH21 isolate, adsorption in 1647 cm^−1^ indicated CO–N, depending on amide groups^45^, and adsorption in the range of 1072–1099 cm^−1^ corresponded to C–O–C bond in the polysaccharide. The adsorption band in 1124 cm^−1^ represented the alkyl functional group (C–C) in the aliphatic chain. In the surfactant produced by SHA302 isolate, the adsorption band in the range of 2300–2400 cm^−1^ could be associated with C–N bonding, and the adsorption band in 1650 cm^−1^ corresponded to a bond between amides I and II, which represent peptide in protein^46^. Moreover, adsorption in 1636 cm^−1^ indicated the carbonyl functional group (COO–)^47^. The absence of adsorption spectrum in the range of 1773–1712 cm^−1^ indicated the absence of lipids in the structure of the emulsifier produced by the SH72 isolate^44^. However, identifying the three biological compounds by TLC and FTIR implied that the biosurfactants and bioemulsifiers produced by SH21, SHA302, and SH72 isolates had glycolipoprotein, lipopeptide, and glycoprotein natures, respectively. The results of this study were consistent with the findings of Refs.^23,48–50^.

### Ionic nature

The ionic nature of the biosurfactants and the bioemulsifier in the absence of a precipitation line towards SDS and at the presence of a precipitation line towards barium chloride indicated the anionic nature of all the three compounds (Fig. S4). Reference^50^ obtained similar results in their study on the ionic nature of glycolipopeptide biosurfactant produced by *L. acidophilus*. The results of ion charge assay in lipoprotein biosurfactant were also consistent with the results reported by Ref.^51^.

### The biosurfactants adsorption to the soil

**Figure 3:**
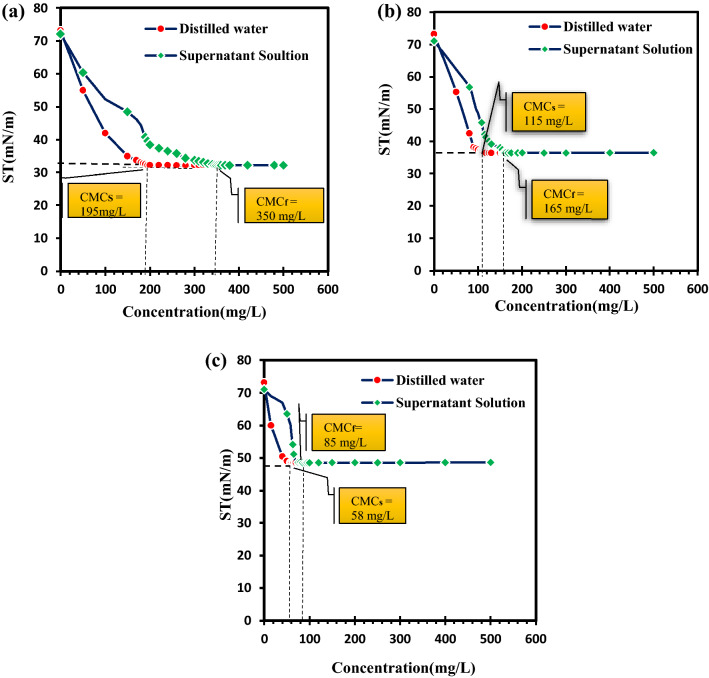
(**A,B**) represent the adsorption of produced biosurfactants into the contaminated soil by SH21 and SHA302, respectively, in different concentrations. (**C**) Adsorption of produced bioemulsifier into the contaminated soil by SH72 in different concentrations. *CMC*_*s*_ measured CMC in distilled water before adding it to the soil, *CMC*_*f*_ measured CMC after adding the surfactant to the soil (adsorption of surfactant to the soil is obtained from the difference between these two CMC).

Due to the adsorption of surfactant monomers into the soil, the probability of micelle formation is reduced and, eventually, the process of contaminants solubilization is limited. Since the adsorption of surfactant into the soil increases its hydrophobicity, petroleum hydrocarbons are reabsorbed from the soluble phase into the soil particles^52^; however, this is due to the extreme hydrophobicity of petroleum hydrocarbons^53^. Reference^54^ accordingly reported that the adsorption of surfactants to the soil reduced their effectiveness. Relatively high adsorption of the surfactants and emulsifier produced by SH21, SHA302, and SH72 isolates into the soil (44.3, 30.3 and 31.8 (%)) could be responsible for the decrease in TPH release efficiency (Fig. 3a–c). Despite the anionic nature of all three compounds produced by SH21, SHA302, and SH72 isolates, its high adsorption into the soil particles could be attributed to the presence of large amounts of calcium and magnesium in the soil^55^. Reference^12^ showed that the low efficiency of anionic surfactant in the soil washing process was due to the high amount of calcium in the soil, which induced the adsorption of surfactant in the soil.

### The effects of biosurfactants and bioemulsifier on the release of TPH from the soil

**Figure 4:**
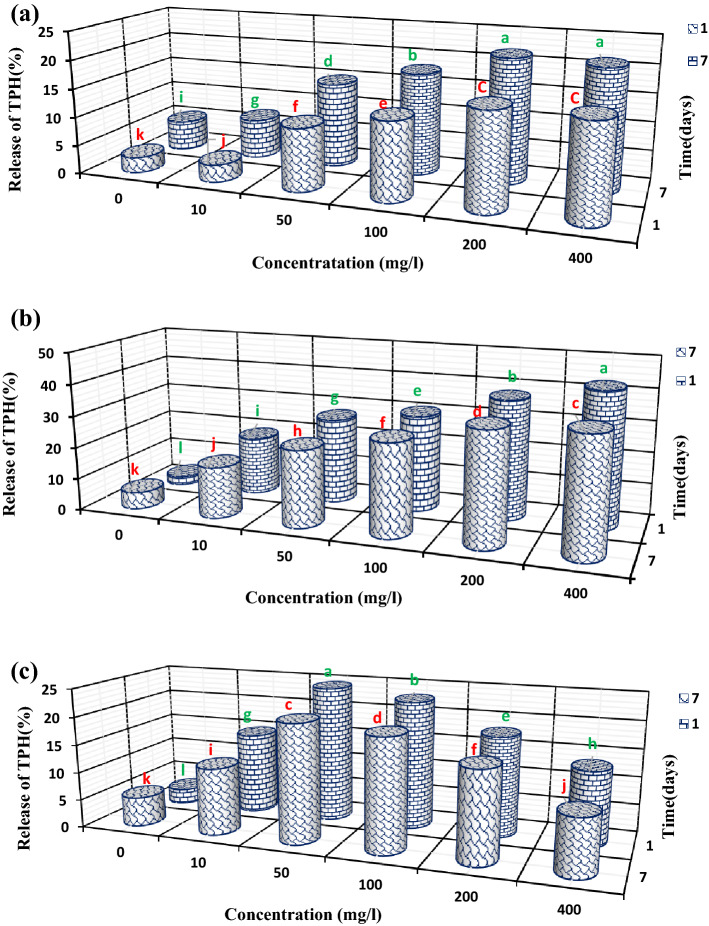
The effects of different concentrations of biosurfactants and bioemulsifier on the release of TPH from the soil at two different times (1- and 7-days). (**a**) Surfactant produced by SH21 isolate, (**b**) surfactant produced by SHA302 isolate, and (**c**) emulsifier produced by SH72 isolate.

Concentration and contact time of biosurfactant/bioemulsifier in and with soil have a crucial effect on removing petroleum compounds from soils^56^. In the present study, the release of TPH from soil was investigated at five different concentrations at intervals of 1- and 7-day. Based on the obtained results, the surfactant produced by the SH21 isolate showed significantly different rates of TPH release from the soil at different concentrations on both day 1 and day 7 compared to the control (distilled water) (Fig. 4a). A significant difference was observed at the biosurfactant concentration of 400 mg/l on days 1 and 7, but this difference was not significant at the concentration of 200 mg/l (≈ CMC) on the same days. The longer the contact time of the biosurfactant with soil, the higher the efficiency of the TPH release from the soil. For the surfactant, as mentioned above, the highest TPH release rate at the CMC concentration (≈ 200) on day 7 was equal to 21.6 ± 0.15%. Reference^57^ studied the effect of lipopeptide surfactant produced by *B. subtilis* SPB1 within 1, 4 and 24 h contact times with soil and showed that it could increase the release of crude oil from the soil over time. From day 1 to day 7, the TPH release rate from the soil showed an increasing trend due to the effective presence of the surfactant and further release of contaminants over time. This could also be attributed to the antibacterial properties of the surfactant and subsequent lack of degradation by soil microorganisms (Fig. S5a, Table 2). The mechanism used by this surfactant for releasing the TPH from the soil might be the mobilization of contaminants. In this surfactant, the contribution of solubilization was minor due to the emulsion’s instability, which could be one of the reasons for reducing the release of the contaminant from the soil (Fig. 2a). The surfactant produced by SHA302 isolate, at all concentrations on both day 1 and day 7, showed significantly different results compared to the control. On day 1, a significant difference in the release of TPH was observed at different concentrations of this surfactant, so that the highest rate of TPH release was obtained at a concentration of 400 mg/l (42.4% ± 0.2). The TPH release rate increased with the increase of this surfactant’s concentration, probably be due to the higher stability of the emulsion formed in the soil solution (Fig. 2b). However, concentrations higher than CMC (200 and 400 mg/l) lead to a significant decrease in TPH release from the soil on both day 1 and day 7. Moreover, by increasing the biosurfactant contact time with the soil, a significant decreasing trend was observed in the release of TPH from the soil (Fig. 4b). Since the biosurfactant produced by the SHA302 isolate had relatively less antibacterial properties than the biosurfactant produced by the SH21 isolate, it was degraded by native soil microorganisms faster (Table 3, Fig. S5b). Therefore, in the long term, it could not affect the soil hydrocarbons and contribute to the release of TPH. Reference^56^ reported a similar result for the release of TPH by surfactin and rhamnolipid at two contact times (1- and 7-day). Re-adsorption of contaminants from soil solution to adsorption sites can be considered another reason for decreasing TPH release over time. However, since the soil is a very complex compound, it is difficult to predict the behaviour of surfactants in releasing organic contaminants from it. However, high levels of Na^+^ within the soil cause swelling and dispersion of clays and slaking of aggregates; as a result, hydraulic conductivity, soil permeability, water, air, and customarily all fluids infiltration are reduced. The swelling phenomenon causes narrowing of the pores, and slaking reduces the quantity of macropores and is followed by a severe decrease in infiltration^58^. Due to a large amount of sodium in the soil under investigation during this study, this biosurfactant’ release efficiency of 42.4% of TPH is fascinating. On the opposite hand, in previous studies, the soils were unnaturally contaminated^57,59^, and though their contamination was natural, the severity of contamination was not excessive^56,60^; however, the soil under investigation during this study has been contaminated for more than sixty years and contains a contamination intensity above 80,000 ppm, and every one of those is essential constraints on soil bioremediation.

**Table 2:** The zone of inhibition diameters (cm) of the studied soil bacteria by the surfactant produced by the SH21 isolate.

Concentrations	SH11	SH12	SH13	SH14
1/2 × CMC	0.56 ± 0.06	0.24 ± 0.02	0.64 ± 0.02	0.25 ± 0.03
CMC	0.73 ± 0.03	0.35 ± 0.01	0.77 ± 0.03	0.27 ± 0.05
2 × CMC	1.35 ± 0.05	0.66 ± 0.02	1.58 ± 0.03	0.84 ± 0.03
4 × CMC	1.8 ± 0.06	142 ± 0.02	1.91 ± 0.06	1.6 ± 0.08

**Table 3:** The zone of inhibition diameters (cm) of the studied soil bacteria by the surfactant produced by the SHA302 isolate.

Concentrations	SH11	SH12	SH13	SH14
1/2 × CMC	–	–	–	–
CMC	0.12 ± 0.01	0.11 ± 0.02	0.12 ± 0.02	0.12 ± 0.02
2 × CMC	0.23 ± 0.01	0.25 ± 0.03	0.31 ± 0.01	0.19 ± 0.01
4 × CMC	0.32 ± 0.03	0.35 ± 0.03	0.55 ± 0.03	0.24 ± 0.04

The bioemulsifier produced by SH72 isolate, at all the concentrations on both day 1 and day 7, showed significantly different TPH releases compared to the control (Fig. 4c). By using the emulsifier produced by SH72 isolate, the highest rate of TPH release from the soil (23.7 ± 0.46 on day 1) was obtained at CMC concentration (50 mg/l). As time passed, the concentrations above CMC led to a significant decrease in the TPH release from the soil. Moreover, from day 1 to day 7, the rate of TPH release decreased due to the lack of antibacterial properties in this surfactant, which exposed it to more degradation by soil microorganisms (Fig. S5c). The reasons for the low efficiency of this bioemulsifier in the release of TPH could be its unstable emulsion formed in the soil solution (Fig. 2c), and its biodegradation by soil native microorganisms, as it had no antibacterial properties. Reference^61^ reported that the biosurfactant produced by *Pseudomonas aeruginosa* UCP0992 did not show a significant difference in oil release with the increase of concentration. Surfactants are involved in releasing petroleum contaminants from the soil through mobilization and solubilization of contaminants. Mobilization occurs at concentrations lower than CMC by reducing surface and interfacial tension, capillary force and contact angle, while the solubilization process occurs at concentrations higher than CMC by forming micelles^14^. The biosurfactant produced by the SH21 isolate did not show a significant difference in the TPH release at the CMC and concentration of 400 mg/l on both day 1 and day 7. However, at the CMC and concentration of 400 mg/l, a significant difference was observed in the TPH release between the first and the seventh days. The results showed that in the surfactant produced by SH21 isolate, the predominant process for removing petroleum compounds was mobilization because no significant difference was observed in the TPH release at concentrations higher than CMC. On the other hand, the emulsion instability that occurred after increasing the biosurfactant concentration suggested the ineffectiveness of the solubilization process in the release of TPH from the soil. A study^62^ reported that mobilisation was the main mechanism for releasing contaminants from the soil. The graphs for the surfactant produced by the SHA302 isolate indicated an upward trend in the release of contaminants after increasing the biosurfactant concentration to CMC and beyond. This confirmed the involvement of both mobilization and solubilization processes in the removal of contaminants. The predominant process in the bioemulsifier produced by the SH72 isolate was mobilization, because a significant difference was observed in the reduction of the release of petroleum contaminants from the soil with the increase of the bioemulsifier concentration beyond CMC.

### Molecular identification

After a comprehensive study of the release of TPH from the soil by surfactants produced by all three isolates, the SHA302 isolate was selected as efficient isolate for molecular identification due to its higher efficiency in TPH release. The 16S rRNA gene sequencing results signified our strain’s highest affinity (93.98%) with *Bacillus pumilus* strain ATCC 7061 ^(T)^ under the accession number NR_043242. The strain was registered in the gene bank under *Bacillus* sp*.* strain SHA302 ^(T)^ under the accession number OK285074. References^63,64^ “suggested that a 16S rRNA sequence similarity of 98% was considered evidence for separate species and a similarity of less than 93 to 95% indicates that organisms should be in different genera”. It is therefore concluded that strain SHA302 belongs to a new species of bacteria. Given that this gram-positive strain is most closely related to the genus *Bacillus*, but unlike all *Bacillus*, it presents a negative catalase and oxidase test, it is possible that it belongs to a new taxon and needs further investigations.

## Conclusion

Purification of the isolates and screening tests revealed that seven isolates had a high potential for biosurfactant production. Among them, six isolates that had the ability to reduce ST to less than 40 mN/m were subjected to the secondary screening (salinity stability test), and finally, three isolates (SH21, SHA302 and SH72) were selected for further analysis. The SH72 isolate did not display a high potential for reducing ST and had a remarkable ability to emulsify n-hexane; it was selected as a bioemulsifier. The release of TPH from the soil increased with the increase of the concentration of biosurfactant produced by the SHA302 isolate. Moreover, using the biosurfactant and bioemulsifier produced by SH21 and SH72 isolates, respectively, the TPH release from the soil increased with the increase of their concentrations to the CMC. However, a further increase in the concentrations of the mentioned biosurfactant and bioemulsifier beyond CMC led to a decreasing trend in the TPH release from the soil. All three biological compounds were anionic and showed relatively high adsorption efficiencies in the contaminated soil. However, the biosurfactant produced by SHA302 isolate, with a TPH release efficiency of 42.4% ± 0.2, could be recommended as a highly effective biosurfactant for the remediation of the saline-sodic soils with old contamination compared to the other biosurfactant and bioemulsifier.

## Materials and methods

### Petroleum-contaminated soil

Sampling was conducted in a highly petroleum-contaminated saline-sodic soil with more than 60 years of contamination (from a depth of 0 to 20 cm) around the oil exploitation well in southern Iran. The samples were transferred to the laboratory and stored at 4 °C until analysis. Physicochemical and biological properties of the contaminated soil were measured^65^. The studied soil was considered to be saline-sodic type, according to (Table S1).

### Bacterial isolates and inoculum preparation

Totally, 39 bacterial isolates from ten oil-contaminated areas in southern Iran were used. The bacteria under study were isolated by Ref.^66^. All the bacterial isolates were enriched in the Bushnell Haas (BH) medium along with n-hexadecane as the sole source of carbon. Experiments commenced by preparing the inoculum based on the method by Ref.^67^. The isolates with an initial population of 1 × 10^8^ (2% v/v) were inoculated in the BH medium with glucose (1% w/v) and *n*-hexadecane (1% v/v) as sources of carbon. Culture medium included: ammonium nitrate (1 g/l), potassium dihydrogen phosphate (1 g/l), dipotassium phosphate (1 g/l), magnesium sulfate (0.2 g/l), iron chloride (0.05 g/l) and calcium chloride (0.02 g/l). The carbon sources in the culture medium were sterilized with a filter (pore size 0.22 µm). Before the sterilization process, the pH of the culture medium was adjusted to 7.0 using NaOH 1 N.

### Screening of biosurfactant-producing bacteria

The initial screening for evaluating the biosurfactant-production potential in the 39 isolates was performed through five tests, including surface tension (ST), emulsification index, oil spreading, foaming, and hemolytic activity. After 96 h of incubation (150 rpm, 30 °C), all the tests (except blood hemolysis) were examined by cell-free culture (CFC) (10,000 g, 10 min).

#### Emulsification index

To measure the emulsification index, 2 ml of the CFC was transferred to the test tubes, and 2 ml of *n*-hexane was added to them. The tubes were mixed with a vortex at high speed for two min and kept at room temperature for 24 h. The height of the emulsified layer was measured, and the percentage of emulsifier was calculated according to the following equation^48^.

$$E24\%=\frac{Height \, of \, emulsified \, layer}{ Height \, of \, the \, whole \, liquid}\times 100.$$

#### Oil spreading test

The oil spreading analysis was conducted by first pouring 40 ml of distilled water into a petri dish (150 mm diameter) and then pouring 15 μl of crude oil onto it to form a thin layer of oil on the surface of the distilled water. Additionally, 10 μl of the CFC were gently poured from a suitable height onto a Petri dish’s centre. At the end, the diameter of the clear halo zone was measured as a qualitative indicator of biosurfactant production^68^. Surface tension activity was performed according to the method of Ref.^69^.

#### Foaming test

For this purpose, 10 ml of the CFC was poured into the test tubes, and the tubes were vortexed at high speed for 2 min; then, the foam height above the liquid phase was recorded, and the foaming percentage was calculated according to the following equation^70^.

$$\text{Percentage of foaming }= \frac{Height \, of \, foam}{ Height \, of \, the \, whole \, liquid }\times 100.$$

#### Hemolytic activity

To investigate the hemolytic activity of the cells, the blood agar medium (5% sheep’s blood) was used to culture the bacterial strain. Then, it was incubated at 24 °C for 48 h. The clear zone formed around the colony represents biosurfactant production^71^.

### Assay of biosurfactants stability to salinity

The salinity stability test was considered a secondary screening for the isolates derived from the primary screening. The stability of biosurfactants to salinity was determined according to Ref.^72^. In order to evaluate the stability of biosurfactants, four parameters, including ST, emulsifying activity, Critical Micelle Dilution of broth diluted 10 and 100 times (CMD^−1^ and CMD^−2^) as response variables in the CFC, were examined after half an hour (25 °C, salinity range of 0–10% NaCl).

#### Biosurfactants extraction

Depending on the type of biosurfactant, several methods were considered for their extraction. The produced biosurfactant was extracted in the bacterial CFC obtained from the primary and secondary screening stages using various extraction methods.

#### Biosurfactants/bioemulsifier extraction by solvents

For this purpose, the bacterial suspension was centrifuged (10,000×*g*, 20 min) after 96 h of incubation and passed through 0.22 μm membrane filters. Afterwards, 6 N HCl was added to the supernatant solution, and the final pH of the solution was raised to 2 and kept at 4 °C overnight. The solution was centrifuged at 10,000×*g* for 20 min to collect precipitates^73^. Then, to the whole of the above suspension, different solvents include chloroform/methanol in a ratio of (2:1)^72^, methanol/chloroform/acetone in a ratio of (1:1:1)^73^, and an equal volume of ethyl acetate and suspension (1:1)^74^ were added. These steps were performed twice for further extraction, the organic phase was collected, and the crude biosurfactant was weighed after solvent evaporation. Cold acetone/suspension (1: 1)^75^ and ethanol^76^ were used to extract the bioemulsifier according to the aforementioned method. Considering the polarity of these two solvents, the suspension solution was mixed with cold acetone and ethanol and centrifuged at 10,000×*g* to collect the formed precipitate.

#### Biosurfactants/bioemulsifier extraction by salts

First, the bacterial suspension was centrifuged to remove the cells, and the resulting supernatant was extracted using ammonium sulfate^77^ and zinc sulfate^73^ at 40% (w/v) and kept at 4 °C overnight. Afterwards, the suspension was centrifuged at 10,000×*g* for 20 min to precipitate the bioemulsifier; finally, the supernatant was removed, and the precipitate was collected. Partial purification of the biosurfactants/bioemulsifier was performed by column chromatography^78^.

### Determination of CMC and observation of emulsion structure

The critical micelle concentration (CMC) indicates the biosurfactant concentration at which ST reaches its lowest value and then remains constant. It was estimated by plotting the ST versus biosurfactant concentration^79^. For this purpose, a stock solution of 1 g/l was prepared from each biosurfactant with sterile distilled water. Afterwards, ST in each concentration was measured in three replications. Observation of the emulsion structure of biosurfactants was done with a slight modification^80^; for this purpose, images of emulsion droplets were obtained using a light microscope (Carl Zeiss-Germany) with a magnification of 40 times. Concentrations of 1/2 × CMC, CMC, and 2 × CMC were prepared from the extracted biosurfactants. After 24 h, 60 μl of an emulsion consisting of crude oil/biosurfactants (1% v/v) was transferred into a cavity slide, and the diameter of the emulsion droplets was measured using a graduated ocular.

### Analysis of biosurfactants by TLC and FTIR

Initial detection of biosurfactants was performed using thin-layer chromatography (TLC) (German Merck, dimensions 6.5 × 2.5 cm). Biosurfactants (0.1 g) were dissolved in methanol, and then 20 μl of the solution was spotted on the TLC plate. Chloroform**/**methanol**/**acetic acid (65:15:2 v/v/v) was used as the mobile phase. Finally, spots were identified with ninhydrin (1%), molisch and iodine vapour reagents^81^. For more accurate results, the identification of biosurfactants was also performed by Fourier transform infrared spectroscopy (FTIR)^48^.

### Determination of biosurfactants ionic nature

First, three wells were created at equal intervals on the surface of a plate containing agarose (1%). The middle well was filled with biosurfactant solution, and the other two wells were filled with 50 mM barium chloride solution and 20 mM sodium dodecyl sulfate solution (SDS). The appearance of a precipitation line between the wells indicated the ionic nature of the studied biosurfactants^51^.

### Measurement of biosurfactants adsorption to the soil

Different concentrations of biosurfactants were poured into the distilled water, and ST was measured at each biosurfactant concentration to obtain the CMC_s_. Afterwards, the prepared biosurfactants concentrations were added to the contaminated soil with a biosurfactant to soil ratio of 2:1, shaken for half an hour (25 °C, 120 rpm) and centrifuged (3000×*g*). ST in the supernatant was measured to obtain CMC_f_^82^. The percentage of biosurfactants adsorption to the soil was measured using Eq. (1).

1 $${\text{X }} = {\text{ CMC}}_{{\text{s}}} {-}{\text{ CMC}}_{{\text{f}}} /{\text{CMC}}_{{\text{f}}} \times { 1}00.$$

### Investigation of biosurfactants antibacterial properties

Four isolates (microbial collection of the University of Tehran, Iran) were cultivated on the surface of agar plates. The wells were created at regular intervals and filled with different biosurfactant concentrations (1/2 × CMC, CMC, 2 × CMC, and 4 × CMC). Distilled water was used as a control. The plates were incubated (24 h, 30 °C), and the zone of inhibition diameters in each concentration was measured with three replications^83^.

### The effect of biosurfactants on releasing TPH from soil

The potential of biosurfactants to release total petroleum hydrocarbons (TPH) was investigated^56^. Different concentrations of the desired biosurfactants/bioemulsifier (10, 50, 100, 200 and 400 mg/l; solution volume: 10 ml) were investigated on the 1st and 7th days (biosurfactant concentrations were determined according to CMC and/or close to it). Briefly, 5 g of dry soil was mixed with 10 ml of the surfactant solutions (ratio 1:2) in different concentrations and shaked (25 C°, 50 rpm). Dry soil was washed with the surfactant solutions, and at two different times, the amount of released TPH from the soil was measured using the gravimetric method^84^. Distilled water was used as a control.

### Molecular identification of isolates

The strain which produced the biosurfactant with the highest efficiency in releasing TPH was subjected to biochemical and molecular identification. Several biochemical analyzes were conducted before the molecular identification, according to Ref.^85^. Qiagen kit Cat. No. 51504 was used to extract the total genomic DNA of the bacterial strain in molecular analysis. The 16S rRNA gene of the pure isolate was amplified, and then the purified PCR products were sequenced by Microsynth Company (Balgach, Switzerland). After editing the sequencing results, the similarity between the obtained sequence and other sequences of known bacteria was determined using the BLAST database. Finally, the identified isolate was recorded in the GenBank.

### Statistical analysis of data

This study was performed as a factorial experiment in a complete randomized design with three replications. The mean values and standard deviation (mean ± SD) were calculated and tested. Two-way analysis of variance (ANOVA) and comparison of mean were carried out using SPSS Statistic 26 software. Probability levels of 0.05 were considered statistically significant. Microsoft Excel software 2013 was used to draw the graphs.

## Supplementary Information

Supplementary Information 1.

Supplementary Information 2.
