# Supplementary material for: The release of petroleum hydrocarbons from a saline-sodic soil by the new biosurfactant-producing strain of Bacillus sp
Source: Sci Rep. 2022 Nov 17;12:19770. doi: 10.1038/s41598-022-24321-3 (PMC9672099; doi:10.1038/s41598-022-24321-3)
Supplement: Supplementary file 1 — Supplementary Information 1. [file 41598_2022_24321_MOESM1_ESM.docx]

| Isolates | Gram Strain | Hemolysis | Emulsification  (%) | Foaming  (%) | Surface Tension  (mN/m) | Oil Spreading |
| --- | --- | --- | --- | --- | --- | --- |
| **SH21** | + | 2.8 | 52/75 | 25/2 | 32/6 | 2.0 |
|  |  | 2.65 | 52/42 | 25 | 32/86 | 1.95 |
|  |  | 2.85 | 52/92 | 25/42 | 33/12 | 1.95 |
| **SH41** | - | 1.5 | 25/52 | 48/31 | 35/63 | 1.65 |
|  |  | 1.75 | 25/3 | 48/12 | 36/14 | 1.75 |
|  |  | 1.6 | 25/45 | 48/62 | 36/42 | 1.7 |
| **SH42** | + | + | 18/22 | 5/65 | 38/44 | 1.1 |
|  |  |  | 18/45 | 5/16 | 38/12 | 1.2 |
|  |  |  | 18/92 | 5/42 | 38/62 | 1.1 |
| **SHA302** | + | 1.8 | 40/5 | 42/2 | 36/25 | 1.8 |
|  |  | 1.65 | 40/25 | 42/46 | 36/72 | 1.75 |
|  |  | 1.75 | 40/33 | 42/62 | 36/52 | 1.65 |
| **SH72** | + | 1.45 | 85/5 | **-** | 48/36 | 1.9 |
|  |  | 1.35 | 85/62 |  | 48/65 | 1.85 |
|  |  | 1.5 | 85/42 |  | 48/92 | 1.8 |
| **SH83** | - | 2.85 | 58/6 | 9/52 | 30/15 | 1.45 |
|  |  | 2.8 | 58/78 | 9/28 | 30/82 | 1.4 |
|  |  | 2.7 | 58/92 | 9/35 | 30/45 | 1.35 |
| **SH103** | - | 2.4 | 22/36 | 15/4 | 28/28 | 1.95 |
|  |  | 2.3 | 22/42 | 15/62 | 28/41 | 1.9 |
|  |  | 2.5 | 22/6 | 15/82 | 28/63 | 2.0 |

**Raw data Table 1**

**Raw data Table 2**

|  | *The zone of inhibition diameters (cm) (SH21)* | | | |
| --- | --- | --- | --- | --- |
| Concentration | SH11 | SH12 | SH13 | SH14 |
| 1/2 CMC | 0/5 | 0/25 | 0/62 | 0/24 |
|  | 0/56 | 0/26 | 0/67 | 0/28 |
|  | 0/62 | 0/22 | 0/64 | 0/23 |
| CMC | 0/72 | 0/34 | 0/78 | 0/29 |
|  | 0/77 | 0/37 | 0/73 | 0/31 |
|  | 0/7 | 0/35 | 0/79 | 0/22 |
| 2 CMC | 1/35 | 0/65 | 1/58 | 0/82 |
|  | 1/3 | 0/68 | 1/54 | 0/88 |
|  | 1/41 | 0/64 | 1/61 | 0/84 |
| 4 CMC | 1/85 | 1/42 | 1/86 | 1/62 |
|  | 1/83 | 1/45 | 1/98 | 1/68 |
|  | 1/78 | 1/4 | 1/9 | 1/52 |

**Raw data Table 3**

|  | *The zone of inhibition diameters (cm) (SHA302)* | | | |
| --- | --- | --- | --- | --- |
| Concentration | SH11 | SH12 | SH13 | SH14 |
| 1/2 CMC | - | - | - | - |
| CMC | 0/13 | 0/12 | 0/13 | 0/1 |
|  | 0/12 | 0/09 | 0/14 | 0/13 |
|  | 0/11 | 0/13 | 0/1 | 0/14 |
| 2 CMC | 0/22 | 0/28 | 0/3 | 0/19 |
|  | 0/25 | 0/25 | 0/31 | 0/2 |
|  | 0/23 | 0/22 | 0/32 | 0/18 |
| 4 CMC | 0/31 | 0/38 | 0/52 | 0/25 |
|  | 0/35 | 0/32 | 0/55 | 0/28 |
|  | 0/3 | 0/36 | 0/58 | 0/2 |

**Raw data Fig 1-a**

| ***Saintly Test for SH21*** | | | | |
| --- | --- | --- | --- | --- |
| % NaCL | ST(mN/m) | E 24(%) | CMD^-1^ | CMD^-2^ |
| 0 | 32.6 | 52/75 | 44/22 | 54/62 |
|  | 32.86 | 52/42 | 44/45 | 54/21 |
|  | 33.12 | 52/92 | 44/52 | 54/86 |
| 1 | 32/82 | 50/54 | 45/8 | 55/42 |
|  | 32/93 | 51/24 | 45/25 | 55/35 |
|  | 33/55 | 51/65 | 45/42 | 55/82 |
| 2 | 33/15 | 48/71 | 46/82 | 56/55 |
|  | 33/28 | 48/92 | 46/54 | 56/12 |
|  | 33/75 | 49/52 | 46/75 | 56/84 |
| 4 | 33/55 | 46/2 | 47/64 | 57/36 |
|  | 33/78 | 47/65 | 47/52 | 57/82 |
|  | 34/2 | 47/32 | 47/35 | 58/1 |
| 6 | 34/12 | 45/2 | 48/12 | 60/2 |
|  | 34/78 | 45/62 | 48/25 | 60/52 |
|  | 35/1 | 46/3 | 48/64 | 60/32 |
| 8 | 35/85 | 44/58 | 49/52 | 62/72 |
|  | 35/55 | 44/32 | 49/21 | 62/4 |
|  | 36/24 | 45/42 | 49/4 | 62/18 |
| 10 | 36/82 | 42/5 | 50/3 | 65/3 |
|  | 36/58 | 42/32 | 50/21 | 65/82 |
|  | 36/9 | 41/82 | 50/48 | 65/54 |

**Raw data Fig 1-b**

| ***Saintly Test for SHA302*** | | | | |
| --- | --- | --- | --- | --- |
| % NaCL | ST(mN/m) | E 24(%) | CMD^-1^ | CMD^-2^ |
| 0 | 36/25 | 40/5 | 47/5 | 54/1 |
|  | 36/72 | 40/25 | 47/23 | 54/33 |
|  | 36/52 | 40/33 | 47/35 | 54/66 |
| 1 | 36/92 | 39/2 | 48/22 | 55/45 |
|  | 36/8 | 40/85 | 48/65 | 55/62 |
|  | 36/85 | 40/66 | 48/54 | 55/82 |
| 2 | 37/15 | 38/65 | 49/1 | 56/22 |
|  | 37/12 | 38/8 | 49/33 | 56/32 |
|  | 37/42 | 38/92 | 49/64 | 56/85 |
| 4 | 37/62 | 37/32 | 50/21 | 57/12 |
|  | 37/82 | 37/51 | 50/13 | 57/63 |
|  | 37/75 | 37/3 | 49/82 | 57/34 |
| 6 | 38/24 | 35/62 | 51/2 | 58/3 |
|  | 38/4 | 35/51 | 51/45 | 58/77 |
|  | 38/62 | 35/85 | 51/62 | 58/92 |
| 8 | 39/32 | 34/32 | 51/9 | 59/36 |
|  | 39/85 | 34/25 | 51/85 | 59/62 |
|  | 39/52 | 34/85 | 52/32 | 59/62 |
| 10 | 40/32 | 33/85 | 52/75 | 60/25 |
|  | 40/65 | 33/32 | 52/9 | 60/52 |
|  | 40/55 | 33/62 | 52/64 | 60/45 |

**Raw data Fig 1- c**

| ***Saintly Test for SH72*** | | | | |
| --- | --- | --- | --- | --- |
| % NaCL | ST(mN/m) | E 24(%) | CMD^-1^ | CMD^-2^ |
| 0 | 48/36 | 85/5 | 52/3 | 62/25 |
|  | 48/65 | 85/62 | 52/41 | 62/5 |
|  | 48/92 | 85/42 | 52/65 | 62/32 |
| 1 | 49/2 | 84/32 | 53/26 | 63/21 |
|  | 49/12 | 84/62 | 53/6 | 63/48 |
|  | 49/35 | 84/95 | 52/93 | 63/95 |
| 2 | 49/85 | 82/85 | 54/85 | 64/4 |
|  | 49/64 | 83/42 | 54/6 | 64/82 |
|  | 49/73 | 83/62 | 54/2 | 64/54 |
| 4 | 50/38 | 82/35 | 55/2 | 65/65 |
|  | 50/21 | 81/8 | 55/36 | 65/14 |
|  | 50/1 | 81/62 | 55/75 | 65/8 |
| 6 | 50/54 | 79/25 | 55/95 | 66/32 |
|  | 50/62 | 79/8 | 56/22 | 66/41 |
|  | 50/95 | 78/82 | 56/82 | 66/2 |
| 8 | 51/21 | 75/62 | 58/25 | 67/25 |
|  | 51/48 | 75/2 | 58/14 | 67/12 |
|  | 51/83 | 75/34 | 58/66 | 68/4 |
| 10 | 52.0 | 73/54 | 59/36 | 68/9 |
|  | 52/25 | 73/52 | 59/62 | 69/52 |
|  | 52/55 | 72/36 | 59/1 | 68/6 |

**Raw data Fig 3- A**

| **Concentration**  **(mg/l)** | **ST(water)**  **(mN/m)** | **ST(supernatant solution)** |
| --- | --- | --- |
| 0 | 72/3 | 72/1 |
|  | 72/2 | 72/1 |
|  | 72/1 | 72/2 |
| 50 | 55 | 60/4 |
|  | 55/1 | 60/8 |
|  | 54/8 | 60/2 |
| 100 | 42/1 | 52/3 |
|  | 41/8 | 52/2 |
|  | 42/3 | 52/4 |
| 150 | 35.0 | 48/5 |
|  | 34/8 | 48/4 |
|  | 35/2 | 48/6 |
| 170 | 33/8 | 46/2 |
|  | 33/5 | 46/3 |
|  | 34/2 | 46/1 |
| 180 | 33/1 | 44/3 |
|  | 32/8 | 44/4 |
|  | 33/3 | 44/2 |
| 185 | 33/4 | 42/3 |
|  | 32/8 | 42/4 |
|  | 32/1 | 42/2 |
| 190 | 32/6 | 41/2 |
|  | 32/5 | 41/3 |
|  | 32/4 | 41/5 |
| 195 | 32/2 | 39/6 |
|  | 32/1 | 39/7 |
|  | 32/4 | 39/5 |
| 200 | 32/2 | 38/4 |
|  | 32/3 | 38/2 |
|  | 32/2 | 38/2 |
| 220 | 32/2 | 37/4 |
|  | 32/2 | 37/5 |
|  | 32/2 | 37/3 |
| 240 | 32/1 | 36/5 |
|  | 32/2 | 36/4 |
|  | 32/3 | 36/6 |
| 260 | 32/4 | 35/8 |
|  | 32.0 | 35/8 |
|  | 32/2 | 35/7 |
| 280 | 32/1 | 34/2 |
|  | 32/2 | 34/4 |
|  | 32/3 | 34/3 |
| 300 | 32/2 | 33/8 |
|  | 32/4 | 33/7 |
|  | 32.0 | 33/9 |
| 310 | 32/2 | 33/5 |
|  | 32/2 | 33/5 |
|  | 32/2 | 33/5 |
| 320 | 32/4 | 33/2 |
|  | 32/1 | 33/1 |
|  | 32/1 | 33/3 |
| 330 | 32/3 | 33.0 |
|  | 32/2 | 32/5 |
|  | 32/1 | 32/8 |
| 340 | 32/4 | 32/2 |
|  | 32/2 | 32/2 |
|  | 32.0 | 32/7 |
| 345 | 32/1 | 32/2 |
|  | 32/2 | 32/6 |
|  | 32/3 | 32/1 |
| 350 | 32/3 | 32/4 |
|  | 32/1 | 34/2 |
|  | 32/2 | 34.0 |
| 355 | 32/4 | 32/2 |
|  | 32/1 | 32/2 |
|  | 32/1 | 32/2 |
| 360 | 32/2 | 32/1 |
|  | 32/4 | 32/3 |
|  | 32.0 | 32/2 |
| 370 | 32/2 | 32/4 |
|  | 32/1 | 32/1 |
|  | 32/3 | 32/1 |
| 380 | 32/2 | 32/3 |
|  | 32/2 | 32/1 |
|  | 32/2 | 32/1 |
| 420 | 32/1 | 32/4 |
|  | 32/4 | 32/1 |
|  | 32/1 | 32.0 |
| 480 | 32/4 | 32/1 |
|  | 32/1 | 32/2 |
|  | 32/1 | 32/2 |
| 500 | 32/3 | 32/2 |
|  | 32.0 | 32/2 |
|  | 32/3 | 32/1 |

**Raw data Fig 3- B**

| **Concentration**  **(mg/l)** | **ST(water)**  **(mN/m)** | **ST(supernatant solution)** |
| --- | --- | --- |
| 0 | 73/1 | 71/5 |
|  | 72/9 | 71/1 |
|  | 72/8 | 71/1 |
| 50 | 55/3 | 62/3 |
|  | 55/2 | 62/4 |
|  | 55/4 | 62/2 |
| 80 | 42/5 | 56/8 |
|  | 41/4 | 56/7 |
|  | 42/6 | 56/8 |
| 90 | 38/2 | 52/6 |
|  | 38/1 | 52/6 |
|  | 38/3 | 52/6 |
| 95 | 37/8 | 50/1 |
|  | 37/6 | 50/1 |
|  | 38 | 50 |
| 100 | 37/4 | 48/6 |
|  | 37/6 | 48/5 |
|  | 37/2 | 48/7 |
| 105 | 37 | 46/2 |
|  | 37 | 46/1 |
|  | 37/1 | 46/3 |
| 108 | 36/8 | 45/2 |
|  | 36/6 | 45/6 |
|  | 37 | 45/4 |
| 110 | 36/7 | 44/4 |
|  | 36/7 | 44/4 |
|  | 36/7 | 44/5 |
| 112 | 36/6 | 43/1 |
|  | 36/6 | 43/2 |
|  | 36/6 | 43 |
| 115 | 36/5 | 42/5 |
|  | 36/4 | 42/4 |
|  | 36/6 | 42/3 |
| 118 | 36/6 | 41/3 |
|  | 36/6 | 41/2 |
|  | 36/5 | 41/4 |
| 125 | 36/6 | 40/1 |
|  | 36/5 | 40/2 |
|  | 36/3 | 40/1 |
| 130 | 36/6 | 39/2 |
|  | 36/4 | 39/1 |
|  | 36/6 | 39/2 |
| 150 | 36/5 | 38/1 |
|  | 36/6 | 38/2 |
|  | 36/5 | 38 |
| 155 | 36/6 | 37/5 |
|  | 36/6 | 37/4 |
|  | 36/4 | 37/3 |
| 158 | 36/6 | 36/5 |
|  | 36/5 | 36/4 |
|  | 36/3 | 36/6 |
| 160 | 36/4 | 36/6 |
|  | 36/6 | 36/6 |
|  | 36/6 | 36/6 |
| 165 | 36/5 | 36/5 |
|  | 36/6 | 36/5 |
|  | 36/4 | 36/6 |
| 168 | 36/6 | 36/5 |
|  | 36/5 | 36/3 |
|  | 36/5 | 36/6 |
| 175 | 36/5 | 36/4 |
|  | 36/5 | 36/6 |
|  | 36/5 | 36/6 |
| 190 | 36/5 | 36/5 |
|  | 36/3 | 36/3 |
|  | 36/6 | 36/6 |
| 200 | 36/5 | 36/6 |
|  | 36/6 | 36/5 |
|  | 36/5 | 36/4 |
| 250 | 36/5 | 36/6 |
|  | 36/5 | 36/5 |
|  | 36/5 | 36/5 |
| 300 | 36/6 | 36/4 |
|  | 36/5 | 36/6 |
|  | 36/4 | 36/6 |
| 400 | 36/5 | 36/6 |
|  | 36/3 | 36/5 |
|  | 36/6 | 36/3 |
| 500 | 36/5 | 36/5 |
|  | 36/5 | 36/3 |
|  | 36/6 | 36/6 |

**Raw data Fig 3- C**

| **Concentration**  **(mg/l)** | **ST(water)**  **(mN/m)** | **ST(supernatant solution)** |
| --- | --- | --- |
| 0 | 72/8 | 71/5 |
|  | 72/8 | 71/1 |
|  | 72/9 | 71/1 |
| 15 | 60 | 69/1 |
|  | 60/1 | 69 |
|  | 60 | 69 |
| 40 | 50/5 | 67/1 |
|  | 50/4 | 67/1 |
|  | 50/6 | 67 |
| 50 | 49/1 | 63/5 |
|  | 49 | 63/4 |
|  | 49/2 | 63/6 |
| 58 | 48/6 | 60/2 |
|  | 48/5 | 60/1 |
|  | 48/7 | 60 |
| 60 | 48/6 | 57/2 |
|  | 48/6 | 57/4 |
|  | 48/7 | 57 |
| 63 | 48/6 | 54/2 |
|  | 48/5 | 54/2 |
|  | 48/4 | 54/3 |
| 65 | 48/6 | 51/1 |
|  | 48/5 | 51/2 |
|  | 48/7 | 51/2 |
| 68 | 48/6 | 49/5 |
|  | 48/6 | 49/4 |
|  | 48/7 | 49/5 |
| 70 | 48/6 | 48/8 |
|  | 48/6 | 48/7 |
|  | 48/6 | 48/8 |
| 75 | 48/6 | 48/6 |
|  | 48/6 | 48/9 |
|  | 48/6 | 48/7 |
| 80 | 48/6 | 48/6 |
|  | 48/7 | 48/8 |
|  | 48/6 | 48/7 |
| 85 | 48/6 | 48/6 |
|  | 48/5 | 48/5 |
|  | 48/6 | 48/6 |
| 90 | 48/8 | 48/6 |
|  | 48/1 | 48/7 |
|  | 49 | 48/5 |
| 95 | 48/6 | 48/6 |
|  | 48/8 | 48/5 |
|  | 48/4 | 48/6 |
| 100 | 48/5 | 48/6 |
|  | 48/7 | 48/5 |
|  | 48/5 | 48/7 |
| 120 | 48/6 | 48/8 |
|  | 48/8 | 48/4 |
|  | 48/6 | 48/6 |
| 150 | 48/6 | 48/5 |
|  | 48/6 | 48/7 |
|  | 48/6 | 48/6 |
| 200 | 48/6 | 48/7 |
|  | 48/6 | 48/5 |
|  | 48/6 | 48/6 |
| 250 | 48/6 | 48/6 |
|  | 48/6 | 48/6 |
|  | 48/6 | 48/7 |
| 300 | 48/6 | 48/8 |
|  | 48/6 | 48/6 |
|  | 48/6 | 48/4 |
| 400 | 48/6 | 48/6 |
|  | 48/6 | 48/5 |
|  | 48/7 | 48/6 |
| 500 | 48/6 | 48/8 |
|  | 48/6 | 48/4 |
|  | 48/7 | 48/6 |

| ***surfactants produced from the isolates SH21*** | | | | |
| --- | --- | --- | --- | --- |
| A | B |  |  | AB |
| Time | Concentration | Rep | Release TPH (%) | Interaction |
| 1 | 1 | 1 | 2/5 | 11 |
| 1 | 1 | 2 | 3/1 | 11 |
| 1 | 1 | 3 | 2/7 | 11 |
| 1 | 2 | 1 | 3/1 | 12 |
| 1 | 2 | 2 | 2/5 | 12 |
| 1 | 2 | 3 | 3/5 | 12 |
| 1 | 3 | 1 | 10/8 | 13 |
| 1 | 3 | 2 | 10/6 | 13 |
| 1 | 3 | 3 | 11/2 | 13 |
| 1 | 4 | 1 | 13/5 | 14 |
| 1 | 4 | 2 | 13/2 | 14 |
| 1 | 4 | 3 | 13/8 | 14 |
| 1 | 5 | 1 | 16/5 | 15 |
| 1 | 5 | 2 | 16/8 | 15 |
| 1 | 5 | 3 | 16/4 | 15 |
| 1 | 6 | 1 | 16/1 | 16 |
| 1 | 6 | 2 | 16/4 | 16 |
| 1 | 6 | 3 | 16/6 | 16 |
| 2 | 1 | 1 | 5/2 | 21 |
| 2 | 1 | 2 | 4/8 | 21 |
| 2 | 1 | 3 | 5/6 | 21 |
| 2 | 2 | 1 | 7/6 | 22 |
| 2 | 2 | 2 | 6/2 | 22 |
| 2 | 2 | 3 | 6/9 | 22 |
| 2 | 3 | 1 | 14/5 | 23 |
| 2 | 3 | 2 | 15/1 | 23 |
| 2 | 3 | 3 | 14/8 | 23 |
| 2 | 4 | 1 | 17/5 | 24 |
| 2 | 4 | 2 | 18.0 | 24 |
| 2 | 4 | 3 | 17/8 | 24 |
| 2 | 5 | 1 | 21/8 | 25 |
| 2 | 5 | 2 | 21/5 | 25 |
| 2 | 5 | 3 | 21/6 | 25 |
| 2 | 6 | 1 | 20/8 | 26 |
| 2 | 6 | 2 | 21/3 | 26 |
| 2 | 6 | 3 | 21/2 | 26 |

**Raw data Fig 4- A**

**Raw data Fig 4- A**

| ***surfactants produced from the isolates SHA302*** | | | | |
| --- | --- | --- | --- | --- |
| A | B |  |  | AB |
| Time | Concentration | Rep | Release TPH (%) | Interaction |
| 1 | 1 | 1 | 2/5 | 11 |
| 1 | 1 | 2 | 3/1 | 11 |
| 1 | 1 | 3 | 2/7 | 11 |
| 1 | 2 | 1 | 18/2 | 12 |
| 1 | 2 | 2 | 18/8 | 12 |
| 1 | 2 | 3 | 18/5 | 12 |
| 1 | 3 | 1 | 27/5 | 13 |
| 1 | 3 | 2 | 27/1 | 13 |
| 1 | 3 | 3 | 26/4 | 13 |
| 1 | 4 | 1 | 30/3 | 14 |
| 1 | 4 | 2 | 30/3 | 14 |
| 1 | 4 | 3 | 30/5 | 14 |
| 1 | 5 | 1 | 36/7 | 15 |
| 1 | 5 | 2 | 38/4 | 15 |
| 1 | 5 | 3 | 37/4 | 15 |
| 1 | 6 | 1 | 42/2 | 16 |
| 1 | 6 | 2 | 42/5 | 16 |
| 1 | 6 | 3 | 42/6 | 16 |
| 2 | 1 | 1 | 5/2 | 21 |
| 2 | 1 | 2 | 4/8 | 21 |
| 2 | 1 | 3 | 5/6 | 21 |
| 2 | 2 | 1 | 16/4 | 22 |
| 2 | 2 | 2 | 16/2 | 22 |
| 2 | 2 | 3 | 15/5 | 22 |
| 2 | 3 | 1 | 24/2 | 23 |
| 2 | 3 | 2 | 24/5 | 23 |
| 2 | 3 | 3 | 23/7 | 23 |
| 2 | 4 | 1 | 33/2 | 24 |
| 2 | 4 | 2 | 32/5 | 24 |
| 2 | 4 | 3 | 32/2 | 24 |
| 2 | 5 | 1 | 35/3 | 25 |
| 2 | 5 | 2 | 35/4 | 25 |
| 2 | 5 | 3 | 34/5 | 25 |
| 2 | 6 | 1 | 36/1 | 26 |
| 2 | 6 | 2 | 37/3 | 26 |
| 2 | 6 | 3 | 35/5 | 26 |

| ***surfactants produced from the isolates SH72*** | | | | |
| --- | --- | --- | --- | --- |
| A | B |  |  | AB |
| Time | Concentration | Rep | Release TPH (%) | Interaction |
| 1 | 1 | 1 | 2/5 | 11 |
| 1 | 1 | 2 | 3/1 | 11 |
| 1 | 1 | 3 | 2/7 | 11 |
| 1 | 2 | 1 | 15/2 | 12 |
| 1 | 2 | 2 | 14/3 | 12 |
| 1 | 2 | 3 | 14/4 | 12 |
| 1 | 3 | 1 | 25/1 | 13 |
| 1 | 3 | 2 | 23/3 | 13 |
| 1 | 3 | 3 | 24/5 | 13 |
| 1 | 4 | 1 | 22/8 | 14 |
| 1 | 4 | 2 | 22/1 | 14 |
| 1 | 4 | 3 | 23/5 | 14 |
| 1 | 5 | 1 | 18/6 | 15 |
| 1 | 5 | 2 | 17/2 | 15 |
| 1 | 5 | 3 | 17/8 | 15 |
| 1 | 6 | 1 | 13/2 | 16 |
| 1 | 6 | 2 | 13/5 | 16 |
| 1 | 6 | 3 | 13 | 16 |
| 2 | 1 | 1 | 5/2 | 21 |
| 2 | 1 | 2 | 4/8 | 21 |
| 2 | 1 | 3 | 5/6 | 21 |
| 2 | 2 | 1 | 12/2 | 22 |
| 2 | 2 | 2 | 11/1 | 22 |
| 2 | 2 | 3 | 12/5 | 22 |
| 2 | 3 | 1 | 20/2 | 23 |
| 2 | 3 | 2 | 21/3 | 23 |
| 2 | 3 | 3 | 21/5 | 23 |
| 2 | 4 | 1 | 19 | 24 |
| 2 | 4 | 2 | 20/2 | 24 |
| 2 | 4 | 3 | 20/5 | 24 |
| 2 | 5 | 1 | 16/2 | 25 |
| 2 | 5 | 2 | 15/5 | 25 |
| 2 | 5 | 3 | 16/5 | 25 |
| 2 | 6 | 1 | 10/7 | 26 |
| 2 | 6 | 2 | 9/8 | 26 |
| 2 | 6 | 3 | 9/2 | 26 |

**Raw data table 4-C**

| ***Extraction OF Surfactant for SHA302*** | | |
| --- | --- | --- |
| ***Rep*** | ***Methods*** | ***curd surfactant(gr)*** |
| 1 | Chloroform/methanol | 0/85 |
| 1 | Ethyl acetate | 0/65 |
| 1 | Ethyl acetate /methanol | 0/47 |
| 1 | Acid precipitation | 0/21 |
| 2 | Chloroform/methanol | 0/92 |
| 2 | Ethyl acetate | 0/6 |
| 2 | Ethyl acetate /methanol | 0/42 |
| 2 | Acid precipitation | 0/2 |
| 3 | Chloroform/methanol | 0/98 |
| 3 | Ethyl acetate | 0/68 |
| 3 | Ethyl acetate /methanol | 0/4 |
| 3 | Acid precipitation | 0/15 |
| 4 | Chloroform/methanol | 0/95 |
| 4 | Ethyl acetate | 0/7 |
| 4 | Ethyl acetate /methanol | 0/5 |
| 4 | Acid precipitation | 0/25 |

**Raw data supplementary**

**Raw data supplementary**

| ***Extraction OF Surfactant for SH21*** | | |
| --- | --- | --- |
| ***Rep*** | ***Methods*** | ***curd surfactant(gr)*** |
| 1 | Chloroform/methanol | 0/64 |
| 1 | Ethyl acetate | 0/52 |
| 1 | Ethyl acetate /methanol | 0/58 |
| 1 | Acid precipitation | 0/25 |
| 2 | Chloroform/methanol | 0/58 |
| 2 | Ethyl acetate | 0/42 |
| 2 | Ethyl acetate /methanol | 0/41 |
| 2 | Acid precipitation | 0/26 |
| 3 | Chloroform/methanol | 0/6 |
| 3 | Ethyl acetate | 0/38 |
| 3 | Ethyl acetate /methanol | 0/45 |
| 3 | Acid precipitation | 0/22 |
| 4 | Chloroform/methanol | 0/73 |
| 4 | Ethyl acetate | 0/52 |
| 4 | Ethyl acetate /methanol | 0/31 |
| 4 | Acid precipitation | 0/12 |

| ***Extraction OF Surfactant for SH72*** | | |
| --- | --- | --- |
| ***Rep*** | ***Methods*** | ***curd surfactant(gr)*** |
| 1 | Ethyl acetate | 0/45 |
| 1 | Acetone | 0/35 |
| 1 | Ethanol | 0/3 |
| 1 | Chloroform/methanol/acetone | 0/1 |
| 1 | Ammonium Sulfate | 0/2 |
| 1 | Zinc Sulfate | 0/12 |
| 2 | Ethyl acetate | 0/5 |
| 2 | Acetone | 0/3 |
| 2 | Ethanol | 0/25 |
| 2 | Chloroform/methanol/acetone | 0/08 |
| 2 | Ammonium Sulfate | 0/25 |
| 2 | Zinc Sulfate | 0/09 |
| 3 | Ethyl acetate | 0/58 |
| 3 | Acetone | 0/4 |
| 3 | Ethanol | 0/32 |
| 3 | Chloroform/methanol/acetone | 0/15 |
| 3 | Ammonium Sulfate | 0/19 |
| 3 | Zinc Sulfate | 0/05 |
| 4 | Ethyl acetate | 0/6 |
| 4 | Acetone | 0/3 |
| 4 | Ethanol | 0/25 |
| 4 | Chloroform/methanol/acetone | 0/15 |
| 4 | Ammonium Sulfate | 0/21 |
| 4 | Zinc Sulfate | 0/14 |

**Raw data supplementary**
