# Supplementary material for: The release of petroleum hydrocarbons from a saline-sodic soil by the new biosurfactant-producing strain of Bacillus sp
Source: Sci Rep. 2022 Nov 17;12:19770. doi: 10.1038/s41598-022-24321-3 (PMC9672099; doi:10.1038/s41598-022-24321-3)
Supplement: Supplementary file 2 — Supplementary Information 2. [file 41598_2022_24321_MOESM2_ESM.docx]

| Term | Amount |
| --- | --- |
| Soil texture | Silty loam |
| pH | 7.52 |
| EC (ds/m) | 80.06 |
| Organic matter (%) | 8.85 |
| Total nitrogen (%) | 0.261 |
| Total Phosphorus (%) | 0.024 |
| Exchangeable Sodium (mg/kg) | 32660 |
| Calcium (mg/kg) | 1200 |
| Magnesium (mg/kg) | 729 |
| SAR (mmol/l)^1/2^ | 258.92 |
| Total Petroleum Hydrocarbon (TPH) (mg/kg dry soil) | 82895 |
| Soil bacterial populations (CFU/ml) | 1.4×10^4^ |

The release of petroleum hydrocarbons from a saline-sodic soil by the new biosurfactant-producing strain of Bacillus sp.

Sahar Kalvandi ^a^, Hamidreza Garousin ^a^, Ahmad Ail Pourbabaee ^*a^, Mohsen Farahbakhsh ^a^

Supplementary Materials

Table. S1. Physicochemical and biological petroleum-contaminated soil characteristics

Table. S2. Comparison of different extraction methods of biosurfactants and bioemulsifiers produced by three isolates of SH21, SHA302, and SH72. Means with the same letters are not significantly different at (P ≤ 0.05).

| Isolate | Methods | Weight of extracted biosurfactants (g/l) | Colour of extracted biosurfactants |
| --- | --- | --- | --- |
|  | Chloroform/methanol | 0.63±0.06^a^ | Dark brown to black |
|  | Ethyl acetate | 0.46±0.07^b^ | Dark brown |
| SH21 | Ethyl acetate /methanol | 0.43±0.1^b^ | Dark brown |
|  | Acid precipitation | 0.21±0.06^c^ | Light brown |
|  |  |  |  |
|  |  |  |  |
|  | Chloroform/methanol | 0.92±0.05^a^ | Light brown |
|  | Ethyl acetate | 0.66±0.04^b^ | Dark brown |
| SHA302 | Ethyl acetate /methanol | 0.45±0.05^c^ | Dark brown |
|  | Acid precipitation | 0.2±0.04^d^ | Light brown |
|  |  |  |  |
|  |  |  |  |
|  | Ethyl acetate | 0.53±0.06^a^ | Milky |
|  | Acetone | 0.34±0.04^b^ | Milky |
| SH72 | Ethanol | 0.28±0.03^b^ | Light brown |
|  | Ammonium Sulfate | 0.21±0.03^d^ | White |
|  | Chloroform/methanol/acetone | 0.12±0.02^c^ | Milky |
|  | Zinc Sulfate | 0.1±0.04^d^ | White |


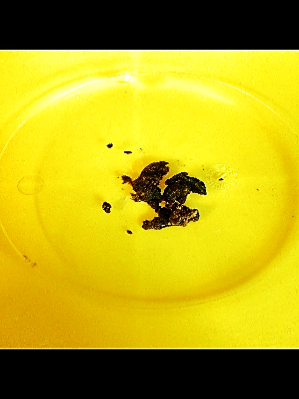
**
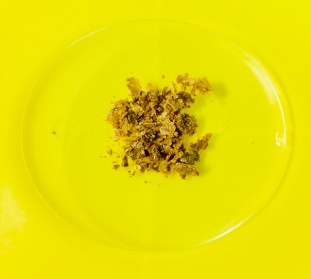

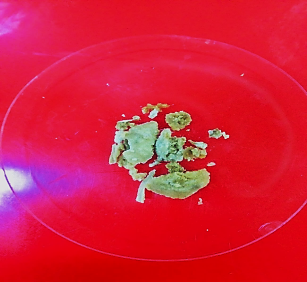
**

### Fig. S1. Raw biosurfactants and bioemulsifier extracted by different methods. A: Surfactant produced by SH21 isolate (chloroform/methanol), B: Surfactant produced by SHA302 isolate (chloroform/methanol), and C: emulsifier produced by SH72 isolate (ethyl acetate/method).

**G**

**F**

**E**

**D**

**C**

**B**

**A**


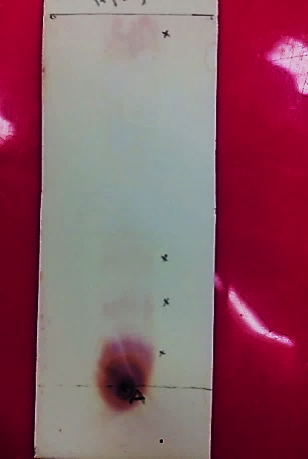

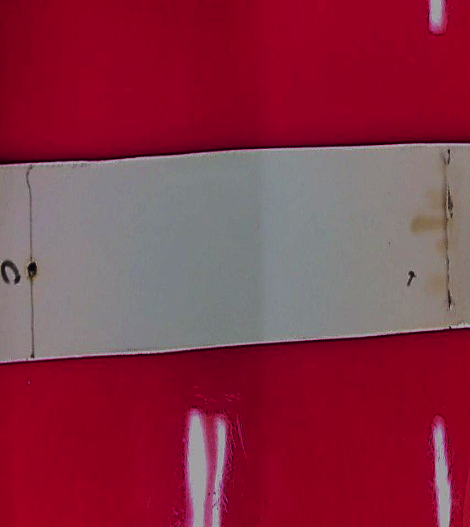

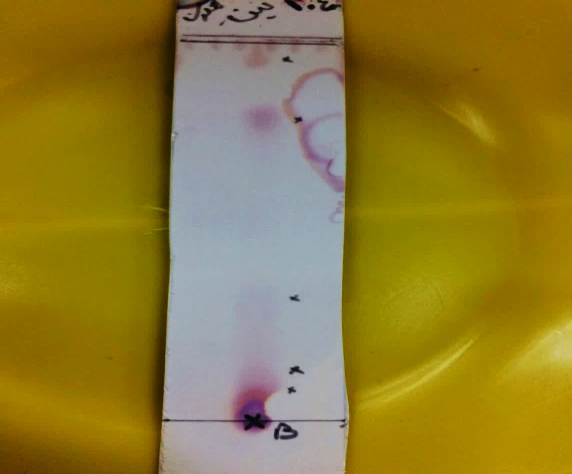

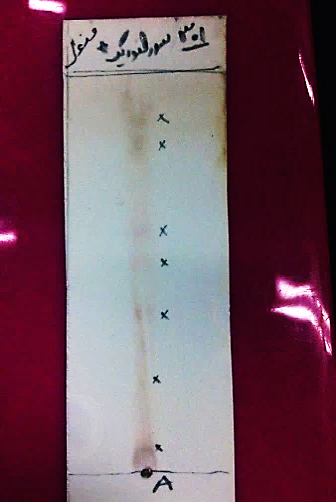
**
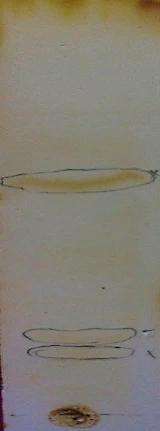
**
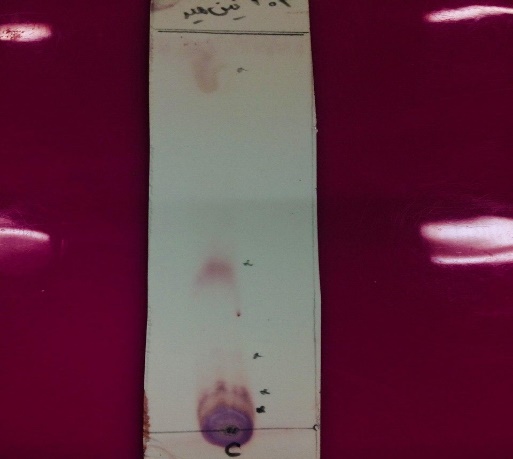
**
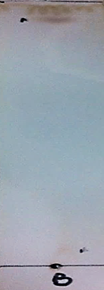
**

Fig. S2. Identification of biosurfactants by TLC. The letters of A and B represent the stains that appeared by the ninhydrin and mulisch reagents, respectively, resulting from the biosurfactant produced by isolate SHA302. C, D, and E letters represent the stains that appeared by the ninhydrin, mulisch and iodine reagents, respectively, resulting from the biosurfactant produced by isolate SH21. The letters of F and G represent the stains that appeared by the ninhydrin and mulisch reagents, respectively, resulting from the bioemulsifier produced by isolate SH72.


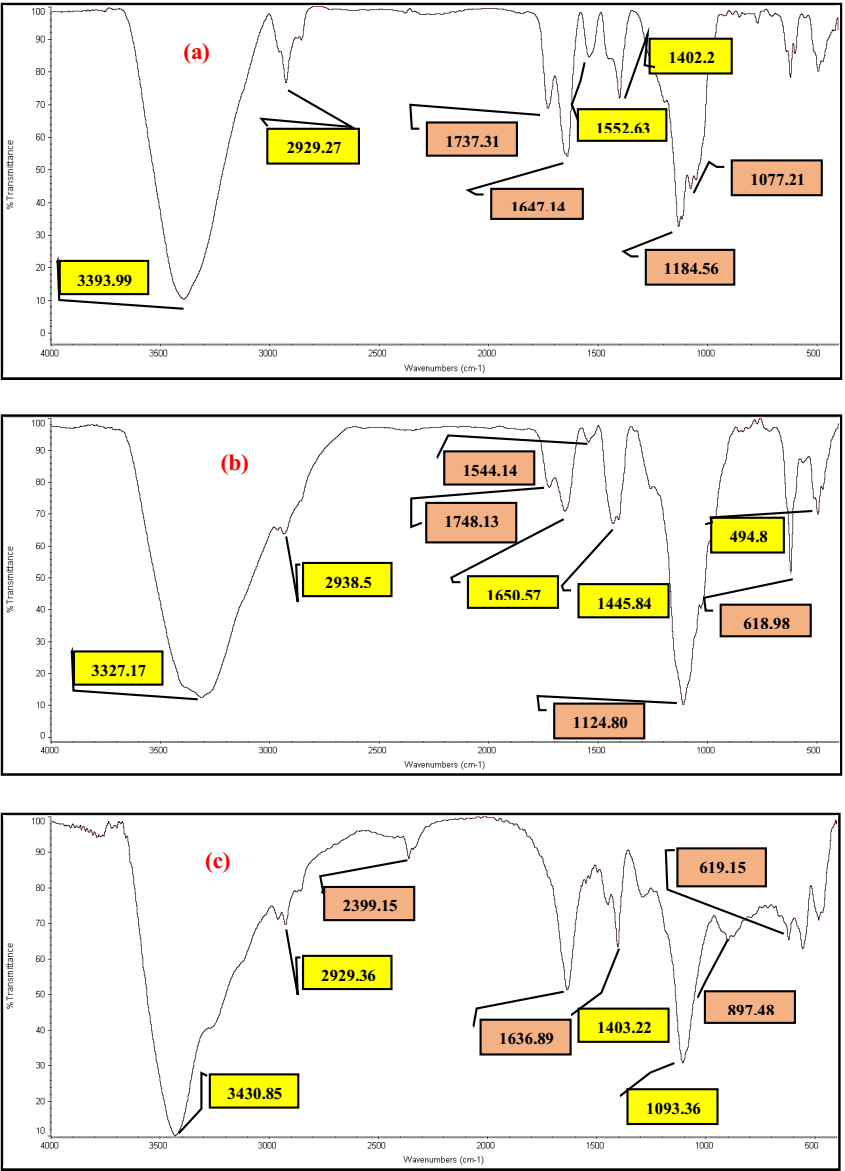


Fig .S3. Fourier Transform Infrared Spectroscopy (FTIR) graphs for biosurfactants and bioemulsifier produced by a: SH21, b: SHA302, and c: SH72.


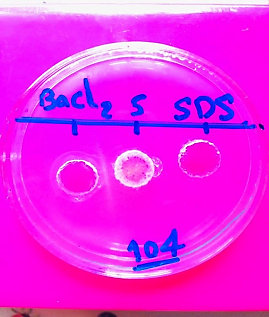

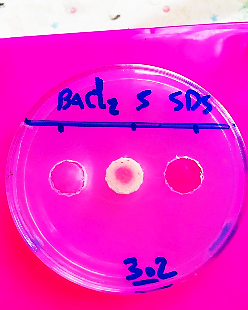

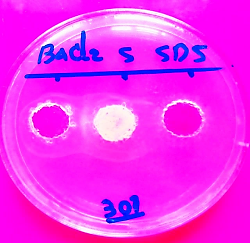


**(a)**

**(c)**

**(b)**

Fig. S4. Ionic nature of biosurfactants and bioemulsifier produced by a: SH21, b: SHA302, and c: SH72

**
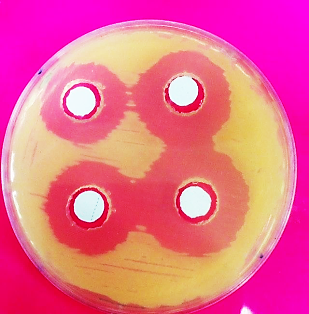

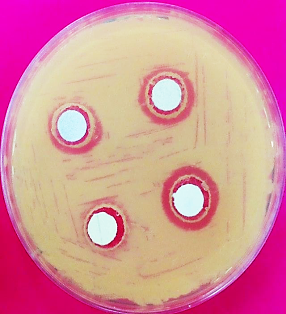
**
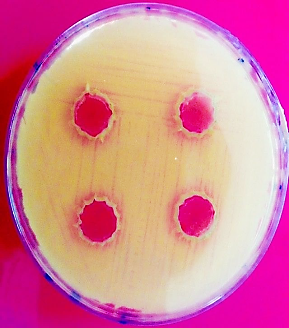


**(c)**

**(a)**

**(b)**

Fig. S5. Antibacterial properties of biosurfactants and bioemulsifier on the selected isolate (SH11). a: surfactant produced by the SH21 isolate, b: surfactant produced by the SHA302 isolate, and c: emulsifier produced by the SH72 isolate.
